# Metacaspase–Peps–PEPR: The 3 musketeers in boosting wheat resistance against *Fusarium* head blight

**DOI:** 10.1093/plcell/koaf182

**Published:** 2025-07-22

**Authors:** Margot Raffeiner

**Affiliations:** Assistant Features Editor, The Plant Cell, American Society of Plant Biologists; Faculty of Biology and Biotechnology, Ruhr University Bochum, Bochum 44801, Germany

Plants truly do not have an easy life. They are exposed to multiple stresses around the clock, including climatic extremes and numerous pathogens. Stressed crops threaten human populations as they result in declining crop yields, jeopardizing food security.

The fungus *Fusarium graminearum* is the main causative agent of *Fusarium* head blight (FHB) disease, which affects one of the most important staple food crops: wheat (*Triticum aestivum* [*Ta*]; Buerstmayr et al. 2020). In addition to annual yield losses restricting wheat consumption, fungicide use to combat FHB disease can harm the environment and human health (Mawcha et al. 2022).

Despite the identification of several hundred resistance quantitative trait loci that can be used for phenotypic plant resistance selection, significant breeding progress for wheat with superior FHB resistance is lacking, since only a few quantitative trait loci have a stable major effect (Mawcha et al. 2022; He et al. 2024). Thus, it is necessary to identify and utilize more effective strategies and targets to control FHB.

Plants' immune responses are induced upon recognition of microbial or self-produced immunogenic compounds, known as pathogen/microbe-associated molecular patterns and phytocytokines, respectively. Pathogen/microbe-associated molecular patterns are conserved molecular structures that are crucial for microbial survival, such as components of the microbial cell wall, membrane, or flagella. Phytocytokines are small signaling peptides whose production is triggered by pathogen infections or environmental stimuli (Hou et al. 2021). They are generated upon cleavage of precursor proteins by proteases and recognized by cell surface–anchored receptor kinases, thereby initiating the mounting of resistance toward different stresses (Hou et al. 2021).

In new work, Yifan Dong and colleagues (Dong et al. 2025) show how plant elicitor peptides (*Ta*Peps), one family of phytocytokines, trigger wheat immunity, thereby boosting resistance against FHB. In silico analysis identified 10 putative precursors of Pep (PROPEPs) in the wheat genome clustered into 3 groups that produce 7 mature *Ta*Peps upon proteolytic processing at conserved residues via *Ta*MCA-IIa (metacaspase), a cysteine protease. By preapplying wheat spikelets with artificially synthesized *Ta*Peps followed by inoculation with *F. graminearum*, the authors showed that group III *Ta*Pep application enhanced wheat FHB resistance, revealing a species-specific role of these Peps in boosting wheat immunity.

While Peps from other plant species can inhibit fungal growth and development and might therefore restrict disease progression, this is not the case for *Ta*Peps. To reveal whether *Ta*Peps confer resistance to FHB via stimulating host immunity, the authors treated wheat spikelets with either *Ta*Peps or the fungal pathogen. They found that *Ta*Pep application induced hallmark plant immune responses, such as the elevation of cytosolic Ca^2+^ concentration, defense gene expression, and mitogen-activated protein kinase signaling, even faster than the pathogen, suggesting that *Ta*Peps function as active immunity inducers. In loss-of-function mutants for a predicted wheat Pep receptor (*Ta*PEPR1), group III *Ta*Peps failed to induce the aforementioned hallmark immune responses or elevate FHB resistance, thereby identifying *Ta*PEPR1 as the receptor for these *Ta*Peps.

To further unravel how *Ta*Peps function in FHB response, the authors infected group III *PROPEP* overexpression (OE) lines and wild type plants with *F. graminearum*. Relative to the wild type, OE lines showed reduced FHB severity and healthier grains concomitant with elevated cytosolic free Ca^2+^ levels, Ca^2+^-dependent mitogen-activated protein kinase phosphorylation, and higher expression of known defense genes.

Last but not least, by using OE and loss-of-function mutants for the wheat metacaspase *Ta*MCA-IIa, the authors identified this protease to be responsible for cleaving *Ta*PROPEPs into functional mature *Ta*PEPs.

Thus, Dong et al. (2025) identified a functional immune module composed of 3 partners that improves wheat FHB resistance (Fig. 1). Importantly, the different OE lines used in this study did not show defects in growth, development, or yield under no-stress growth conditions, suggesting that this tripartite immune module might be exploited to improve wheat FHB resistance without negative pleiotropic effects.

**Figure.**
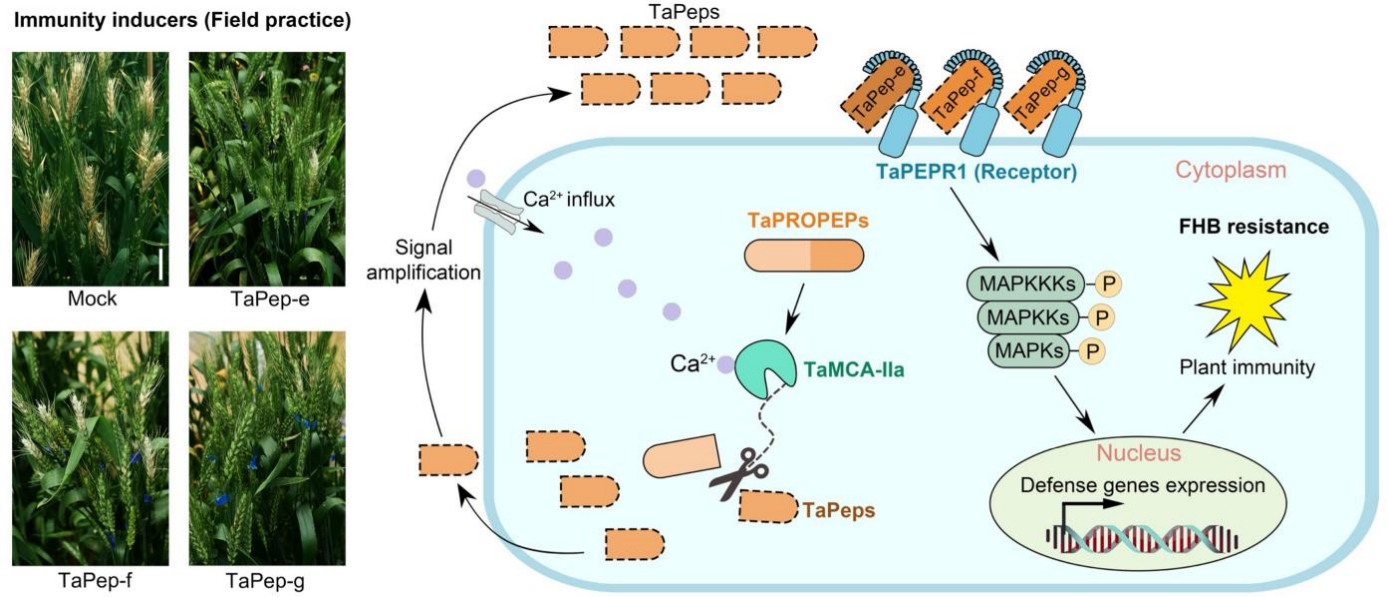
The metacaspase–Peps–PEPR module enhances resistance to FHB. Three *Ta*Peps (*Ta*Pep-e, -f, and -g) function as immunity inducers, boosting wheat resistance to FHB in field conditions (left). The precursors, *Ta*PROPEPs, are cleaved by *Ta*MCA-IIa to generate mature *Ta*Peps, which are recognized by the receptor *Ta*PEPR1, triggering immune signaling and calcium dynamics, thus conferring resistance to FHB (right). Adapted from Dong et al. (2025).

## Recent related articles in *The Plant Cell*

- Liu et al. (2024) review the role of protein degradation mediated by the ubiquitin proteasome system and proteases in regulating the homeostasis of plant immune regulators, including a discussion of phytocytokines.
- Sutherland et al. (2025) review how plant immune receptor diversity can be translated into engineering efforts from *Arabidopsis* to crops.
- Zhang et al. (2024) show that the interaction of the histone acetyltransferase GCN5 with the calmodulin-binding transcription factor CAMTA2 regulates wheat grain size and weight.

## Data Availability

n/a.

## Dive Curated Terms

The following phenotypic, genotypic, and functional terms are of significance to the work described in this paper:

Fusarium Gramene: EO:0007341
Fusarium Araport: EO:0007341
